# Assessment of clinical prognosis improvement in children with concomitant anterior and posterior urethral valves: A case series report

**DOI:** 10.1097/MD.0000000000037004

**Published:** 2024-01-26

**Authors:** Zhiqiang Mo, Weiping Zhang, Ning Sun, Jun Tian, Minglei Li, Hongcheng Song, Xianghui Xie

**Affiliations:** a Urology Department, Shunyi Maternal and Children’s Hospital of Beijing Children’s Hospital, Beijing, China; b Pediatric Medical School, Capital Medical University, Beijing, China; c Urology Department, Beijing Children’s Hospital, Capital Medical University, National Center for Children’s Health, Beijing, China.

**Keywords:** Anterior urethral valves, missed diagnosis, posterior urethral valves, prognosis

## Abstract

**Background:**

Concomitant anterior urethral valves (AUVs) and posterior urethral valves (PUVs) is an extremely rare congenital urologic anomaly, which may be easily overlooked in the clinic.

**Objective:**

This study assessed the prognosis of children with concomitant PUVs and AUVs.

**Methods:**

The clinical data of inpatients with concomitant AUVs and PUVs in our hospital were collected from January 1983 to June 2022. The clinical manifestations, auxiliary inspection, and treatment were described in detail.

**Results:**

In total, 6 cases of concomitant AUVs and PUVs in boys were found in our hospital, with ages ranging from 3 months to 9 years; the main clinical manifestation was abnormal urination. Four patients exhibited concomitant AUVs and PUVs preoperatively and underwent simultaneous anterior and posterior urethral valvotomy. Follow-up studies showed that 3 patients’ clinical symptoms substantially improved with well-maintained renal function. One patient died of renal failure. In the other 2 patients, PUVs were initially identified and excised, but their clinical symptoms did not show substantial improvement. Following voiding cystourethrography (VCUG), the AUVs were found and obstructions were then completely relieved. However, 2 patients died of renal failure.

**Conclusions:**

If urinary symptoms cannot be substantially relieved after posterior urethral valvotomy, VCUG and cystoscopy should be repeated to shorten the interval between anterior and posterior urethral valvotomies to improve patient prognosis.

## 1. Introduction

Posterior urethral valves (PUVs) have an incidence rate of approximately 1/5000 to 1/8000 among male neonates,^[1]^ while anterior urethral valves (AUVs) are less common than PUVs, with an incidence rate of approximately 1/15 to 1/30 relative to that of PUVs.^[2]^ Because it constitutes an extremely rare congenital urologic anomaly, the clinical understanding of concomitant PUVs and AUVs has remained poor.^[3]^ The mechanism of concomitant PUVs and AUVs is unclear, and is presumably similar to that of PUVs, with respect to gene mutations.^[4]^ However, AUVs and PUVs may have entirely different origins^[5,6]^; PUVs might be caused by mutations in the angiotensin enzyme gene,^[7]^ and concomitant PUVs and AUVs may simply be coincidental. Further studies are thus needed to investigate the mechanism of this anomaly.

The clinical symptoms of concomitant PUVs and AUVs mainly consist of abnormal urine flow, urinary tract obstruction, and infection, as well as other manifestations that may occur in children with AUVs or PUVs alone. Jenni and colleagues have reported that achievement of urinary continence in patients with PUVs is greatly delayed when compared with their healthy peers,^[8]^ which may be due to elevated intravesical pressure associated with dysfunctional bladder emptying. Whether the obstruction can be completely and immediately removed will directly affect the children prognosis.

In recent years, the prognosis of patients with PUVs has been greatly improved; however, despite intrauterine intervention, the long-term prognosis of these patients remains poor.^[9]^ Previous studies have revealed that the prognosis of patients with AUVs is slightly better than or similar to that of patients with PUVs.^[10,11]^ Concomitant PUVs and AUVs are typically accompanied by vesicoureteral reflux and renal damage, which exhibit a prognosis similar to that of patients with PUVs.^[12]^ However, due to similarities in clinical manifestations between PUVs and concomitant PUVs and AUVs, it is easy to overlook AUVs, even when examined by voiding cystourethrography (VCUG) or cystoscopy. This initial missed diagnosis leads to increased diagnostic time, continuous damage to the urinary system, and ineffective relief of lower urinary tract obstruction, as well as worse prognosis than PUVs.

Because missed diagnosis of PUVs or AUVs leads to increased diagnostic time and worse treatment outcomes, patients’ prognoses are seriously affected. Thus far, only 18 cases of patients with concomitant PUVs and AUVs have been described in the literature^[5,13–15]^; the largest study involved 6 patients. Several factors may contribute to the missed diagnosis. First, doctors do not consider the concomitant conditions due to lack of knowledge or experience,^[3]^ especially when patients’ symptoms are partially relieved after PUV or AUV incision. Second, doctors do not perform sufficient follow-up, resulting in a lack of understanding regarding subsequent disease manifestations; patients may show slight improvement in symptoms due to incomplete AUV obstruction before PUV incision^[6]^ or temporal improvement of urination caused by extended-duration urethral catheter expansion after PUV incision. Third, VCUG is currently the main method for diagnosis of PUVs or AUVs before cystoscopy. However, Carvell^[16]^ presumed that missed diagnosis by VCUG is related to imaging techniques; because of the presence of PUVs, VCUG can only detect expansion of the posterior urethra and incomplete distal urethral filling, resulting in difficulty diagnosing concomitant PUVs and AUVs when using VCUG alone. In addition, when using cystoscopy, the following factors may lead to missed diagnosis: flattened urethral wall, adhesion of the valves to the urethral wall in retrograde urethrography,^[17]^ and similarity in color between the valves and urethral wall. Thus, we presume that the actual number of patients with concomitant PUVs and AUVs might be more than 18.

## 2. Material and methods

In the present study, we reviewed the medical records of 530 patients with PUVs in our hospital from January 1983 to June 2022, of which 6 patients had concomitant PUVs and AUVs; the clinical data of these 6 patients were analyzed retrospectively.

In total, 6 boys were included in our study, and the age at the first doctor visit ranged from 3 months to 9 years (average of 30 months), which was nearly identical to the age when the patients underwent posterior urethral valvotomy (average of 31 months). Of the 6 patients, 4 underwent anterior and posterior urethral valvotomy simultaneously. Two patients underwent anterior urethral valvotomy 2 months and 1 year after undergoing posterior urethral valve valvotomy. No serious diseases of other systems were found in any of the 6 boys.

## 3. Clinical manifestation

At the time of clinical presentation, symptoms included dysuria, urine, dribbling, frequent urination, nocturnal enuresis, and urinary tract infections. With the exception of the patient who underwent simultaneous PUV and AUV valvotomy, 1 patient who underwent PUV valvotomy in the first operation showed no substantial improvement in symptoms, whereas 1 reported slight relief of dysuria. Abnormal urination remained the main clinical manifestation, with no substantial change postoperatively.

## 4. Auxiliary inspection

**Figure 1.**
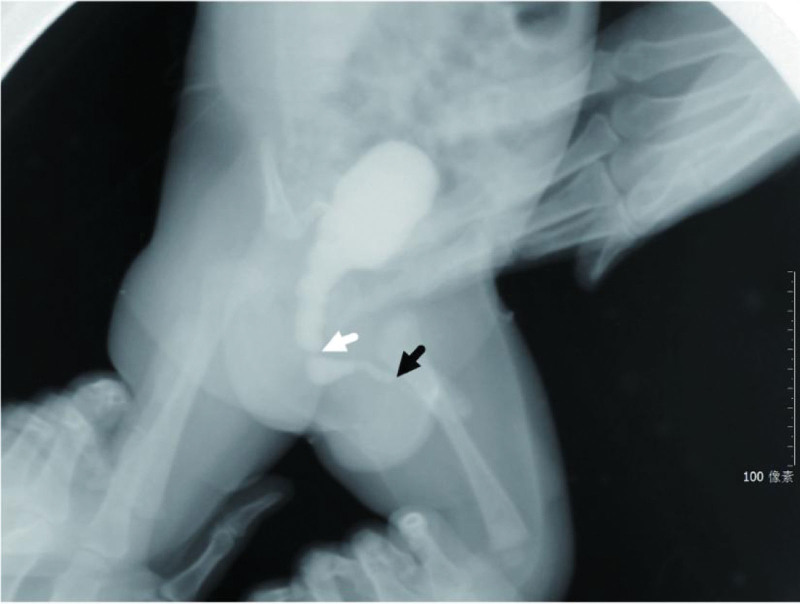
Distal obstruction of the posterior urethra with posterior urethral dilatation (white arrow) and anterior urethral couch obstruction (black arrow) with proximal anterior urethral dilatation before surgery.

**Figure 2.**
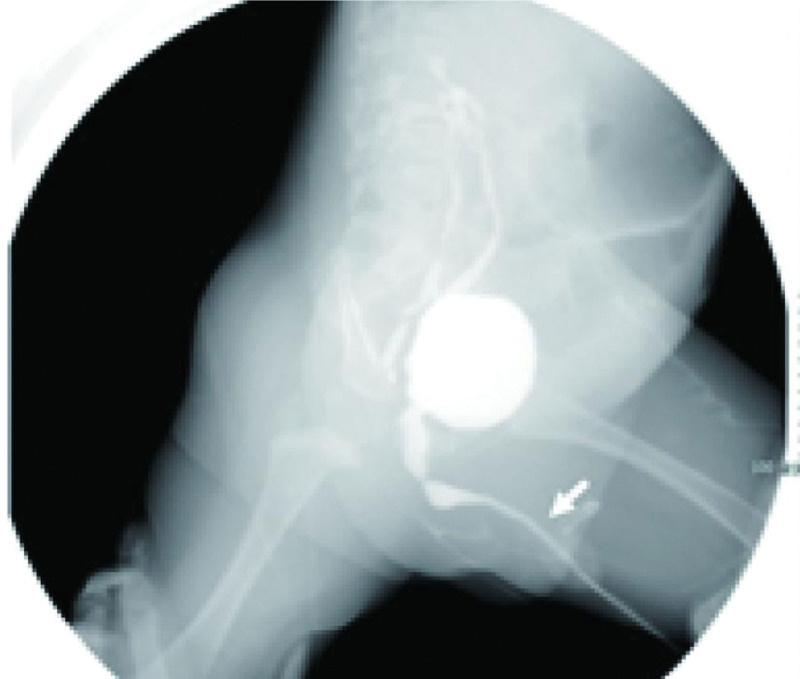
Preoperative examination with dilatation of the posterior (white arrow) and anterior (black arrow) urethras.

VCUG (Figs. 1 and 2), color Doppler ultrasonography, intravenous pyelography, and renal function examination were performed before the first operation in all 4 patients. However, 1 patient completed these examinations in another hospital. All patients were preoperatively diagnosed with PUVs; 4 patients were also preoperatively diagnosed with vesicoureteral reflux. Among the 6 patients, 1 exhibited pop-off (a sign of unilateral reflux and dysplasia), small right kidney, and ipsilateral reflux; 1 patient had sedimentation in the left renal lower calyx, and 1 patient had substantially reduced uniformity of the right kidney. All patients underwent the first cystoscopy, by which 2 patients were diagnosed with PUVs and 4 were diagnosed with concomitant PUVs and AUVs.

**Figure 3.**
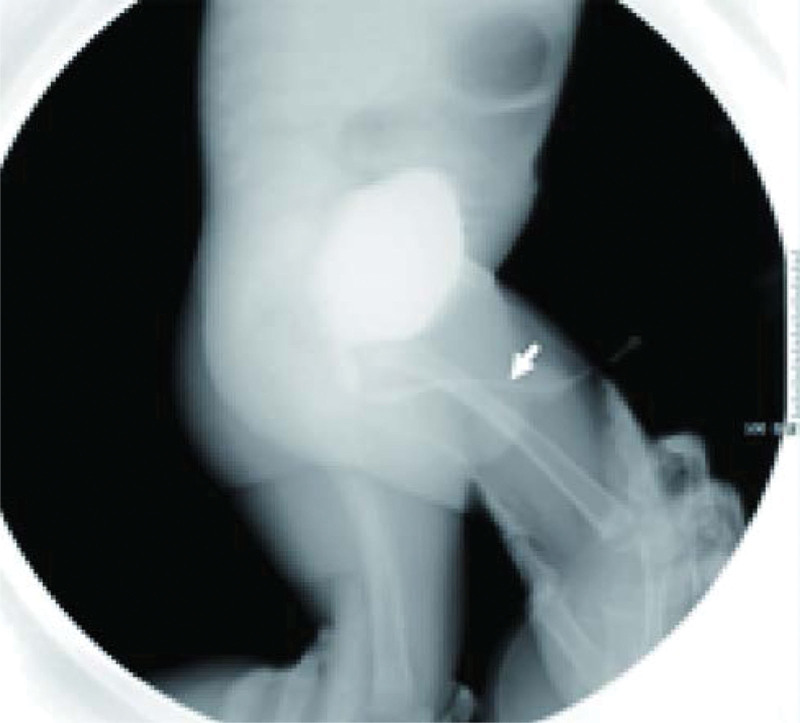
Bilateral vesicoureteral reflux (grade II) after anterior and posterior urethral valvulotomy; the anterior and posterior urethra remained slightly expanded, with unobstructed urethra and clear and strong urine line.

**Figure 4.**
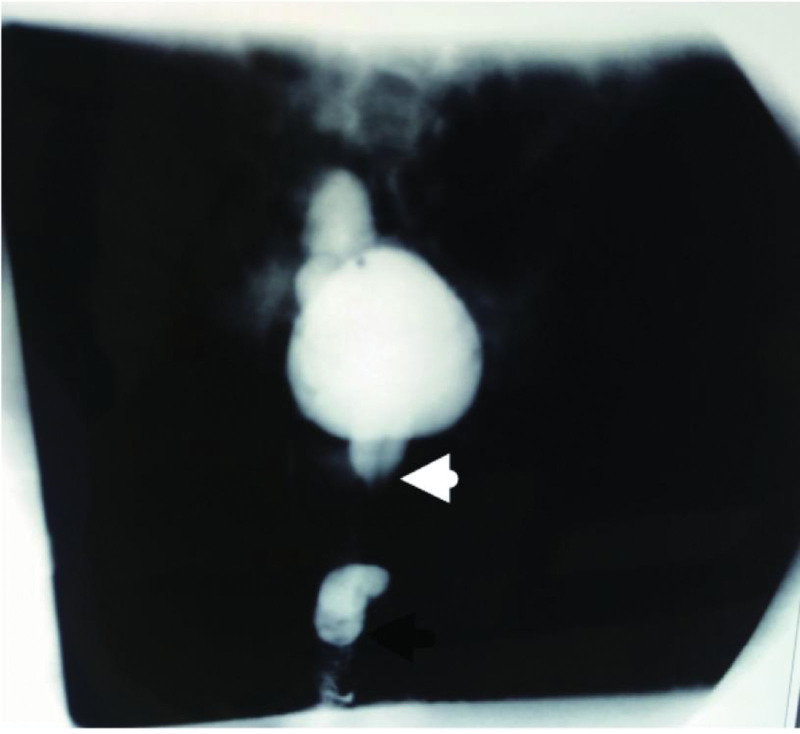
Posterior urethral dilatation was greatly improved after posterior urethral valvulotomy. Couch obstruction and vesicoureteral reflux (grade III) remained visible in the anterior urethra (arrow).

Two patients who underwent PUV valvotomy in the first operation underwent VCUG (Figs. 3 and 4), urinary tract ultrasound, serum creatinine, and other tests before the second surgery. Incomplete anterior urethral obstruction was observed when using VCUG, indicating the existence of AUVs. Ultrasound examination showed mild hydronephrosis and rough bladder walls. In addition, 1 patient had reduced volume in the right kidney and nonuniform renal parenchyma, whereas 1 patient had sedimentation in the right renal lower calyx. No abnormalities were found in serum creatinine and urine protein examinations.

## 5. Treatment

All 6 patients underwent operations by transurethral cystoscopy. Four patients underwent simultaneous anterior and posterior urethral valvotomy. During the operation, a urethral stricture caused by ventral bulge at the penis root was initially observed, and an iris-like valve was then found in the distal verumontanum with a trabecular bladder. After withdrawal of the cystoscope, the AUVs were cut with a metal electric hook. Regarding PUVs that were thinner on both sides of the wall and were more hypertrophic at the 12 o’clock position, PUVs were burned off at 5 o’clock, 7 o’clock and 12 o’clock positions, successively. The other 2 patients, who had undergone initial surgery in other hospitals, exhibited normal bladder capacity, rough bladder mucosa, and completely incised PUVs at the position of verumontanum during the second surgery. The AUVs were found at the root of the penis, and complete incision was performed at the 6 o’clock position. Bladders were squeezed after withdrawal of the cystoscope, and the urine stream was strong in all patients. The catheter was regularly preserved postoperatively.

## 6. Outcomes

**Figure 5.**
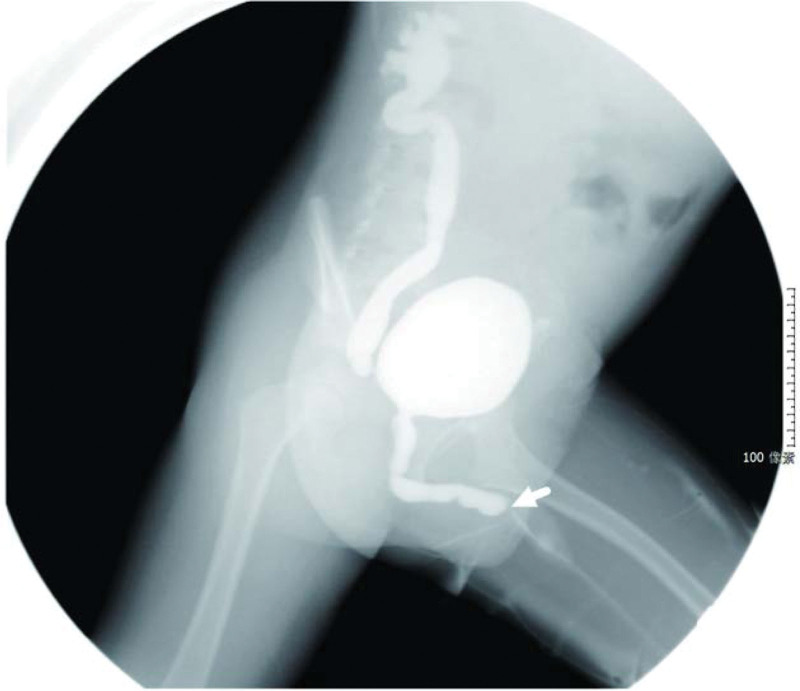
One year postoperatively, bilateral ureteral reflux disappeared and urination was smooth.

**Figure 6.**
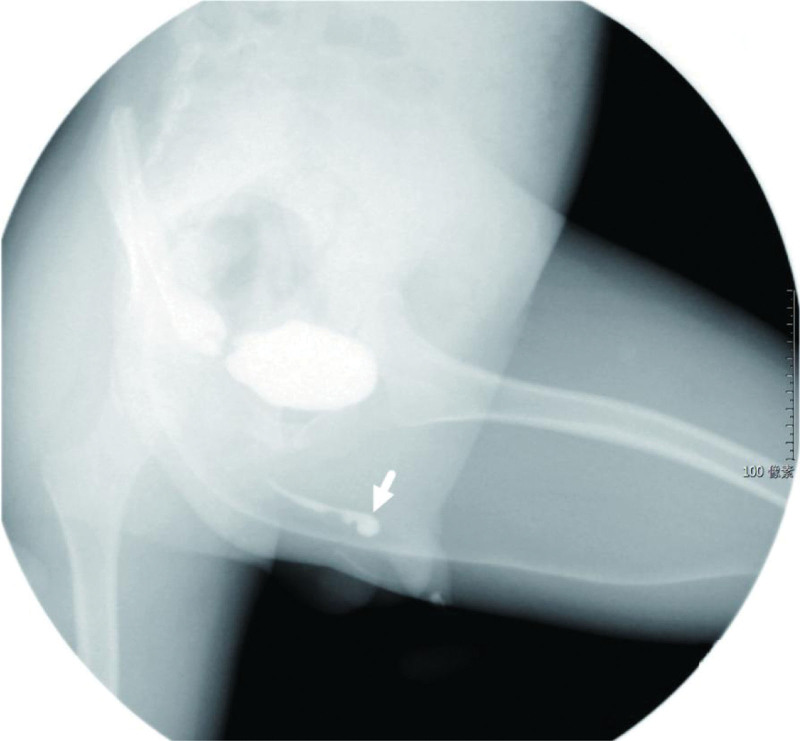
After anterior and posterior urethral valve surgery, ureteral reflux disappeared with limited residual contrast agent at the end of urethral urination.

The catheter was removed at 1 to 2 weeks postoperatively. The follow-up duration was 1 to 26 months, with an average of approximately 17 months. One patient (first visit at the age of 9 years, simultaneously excised the PUVs and AUVs) had recurrent fever with increasing bilateral vesicoureteral reflux postoperatively. Bilateral urethral replantation surgery was performed, but the patient condition continuously worsened and the patient died of renal failure following discharge from the hospital. The remaining 5 patients were followed up after anterior urethral valvotomy and underwent VCUG, urological ultrasound, urodynamic examination, and renal function examination. Two patients who underwent a second valvulotomy died of renal failure about 1.2 years later. Symptoms did not substantially change during follow-up for the 2 patients with mild clinical presentations after surgery, while 1 patient showed mild dysuria and occasional urinary drip. VCUG (Figs. 5 and 6) revealed improved ureter reflux and mild dilatation of the anterior proximal urethra in 2 patients. Ultrasound revealed kidney scars in 2 patients. Urodynamic examination showed stable bladder detrusor at the filling stage; bladder compliance was slightly lower than normal, while detrusor are flexia was normal during the micturition phase.

## 7. Discussion

Previous studies have revealed that patients with concomitant PUVs and AUVs are relatively rare in clinic, comprising 1.1% and 0.02%, respectively, of all PUV cases and inpatients.^[12]^ In our hospital, a total of 530 PUV patients were hospitalized from January 1983 to June 2022, and only 1.13% of patients exhibited concomitant AUVs, which was similar to the findings in other reports.

Early diagnosis of concomitant PUVs and AUVs is extremely important for disease prognosis. Earlier diagnosis of PUVs presumably leads to better patient prognosis. Concomitant PUVs and AUVs are accompanied by vesicoureteral reflux and renal damage, with a prognosis similar to that of PUVs.^[12]^ In the current study, 1 patient first visited doctors after 9 years of repeated enuresis. Although we simultaneously excised the PUVs and AUVs, the patient ultimately progressed to renal failure. In contrast, 3 patients were reviewed early and diagnosed in a timely manner; both had better outcomes. All of these findings suggest that age at first visit greatly affects prognosis. In addition, it has been reported that clinical symptoms are determined by the age at diagnosis for patients with concomitant PUVs and AUVs. Although patient age at the time of first treatment ranged from 3 months to 9 years, no unique symptoms were found in our patients, which may partially be related to our relatively small sample size.

As mentioned above, missed diagnosis will greatly affect patient prognosis. Several measurements may help to reduce missed diagnosis of this disease. First, multiple methods can be used to detect concomitant PUVs and AUVs; in our study, AUVs were not found when using VCUG, ultrasound, and other tests, while cystoscopy detected concomitant PUVs and AUVs in 1 patient before the initial surgery. In addition, as a nonradioactive method, urodynamic examination can reflect bladder function and acts as an important means to evaluate bladder function. Generally, patients should receive urodynamic examination if postoperative urinary symptoms exist. Furthermore, doctors can press the bladder to promote urethral dilatation, then determine whether the obstruction has been completely removed. Second, because the anterior urethra cannot be fully dilated due to PUV obstruction,^[16]^ oblique view VCUG with full-length delineation of the urethra is of paramount diagnostic importance.^[12]^ Similarly, operators should note whether there are valves in the anterior urethra when the cystoscope is withdrawn to the distal urethra. Third, surgeons should fully understand this disease and establish close cooperation with doctors from other departments; in the current study, 1 patient showed slightly improved symptoms postoperatively. If the author had no knowledge of this disease, the patient might have been more likely to receive a missed diagnosis; in addition, the patient with concomitant PUVs and AUVs was finally diagnosed by the author with the aid of radiologists, which suggested the importance of cooperation among doctors. Fourth, follow-up is important to avoid missed diagnosis, even after surgery. In our study, postoperative follow-up helped to evaluate bladder function and detected the existence of persistent mechanical obstruction. Finally, education of parents after PUV surgery is critical.^[18]^ Doctors should inform parents of possible changes in patients’ conditions, as well as precautions after valvotomy. VCUG, cystoscopy, and other tests should be performed again if insufficient improvement is achieved in urological symptoms, which is particularly important in areas with poor economic and sanitation conditions.

Early release of lower urinary tract obstruction is the basic treatment for concomitant PUVs and AUVs. Although incision of the valves may result in poor prognosis, 1 of our patients died postoperatively, which may be related to renal dysplasia, bladder dysfunction, and delayed operation; however, simultaneous or short interval incision remains the basis for improving prognosis. Notably, diagnosis of concomitant PUVs and AUVs may be easily missed, leading to incomplete release of obstruction and poor prognosis. In our study, AUVs were not diagnosed in 2 patients in the preoperative and intraoperative examinations. Based on our experience, we propose that examinations, such as VCUG, urodynamics, and cystoscopy, should be performed if symptoms are not greatly relieved within 1 to 2 weeks after catheter removal. In addition, reports have revealed that angiotensin-converting-enzyme inhibitor drugs can protect renal function and improve the prognosis of patients who have undergone surgery to relieve lower urinary tract obstruction.^[19]^ Appropriate medication may also play roles in improving patient prognosis. However, symptoms may not be improved, despite surgery, in patients with poor bladder function, concomitant urethral stricture, or incomplete valve incision.

## 8. Conclusions

Although concomitant AUVs and PUVs are rarely observed in clinic, missed diagnosis can seriously affect patient prognosis. If urological symptoms are not greatly relieved after posterior urethral valvotomy, concomitant AUVs and PUVs should be considered, followed by VCUG, cystoscopy, and other tests to shorten the interval between anterior and posterior urethral valvotomies, thereby improving patient prognosis.

## Author contributions

**Conceptualization:** Zhiqiang Mo.

**Data curation:** Zhiqiang Mo.

**Project administration:** Zhiqiang Mo.

**Supervision:** Zhiqiang Mo, Weiping Zhang, Ning Sun, Minglei Li, Hongcheng Song, Xianghui Xie.

**Writing—original draft:** Zhiqiang Mo.

**Writing—review & editing:** Xianghui Xie, Weiping Zhang, Ning Sun, Jun Tian.
